# Antenatal Syphilis Screening Using Point-of-Care Testing in Sub-Saharan African Countries: A Cost-Effectiveness Analysis

**DOI:** 10.1371/journal.pmed.1001545

**Published:** 2013-11-05

**Authors:** Andreas Kuznik, Mohammed Lamorde, Agnes Nyabigambo, Yukari C. Manabe

**Affiliations:** 1 Infectious Diseases Institute, Makerere College of Health Sciences, Kampala, Uganda; 2 Pfizer, New York, New York, United States of America; 3 Division of Infectious Diseases, Department of Medicine, Johns Hopkins School of Medicine, Baltimore, Maryland, United States of America; Hospital Clinic Barcelona, Spain

## Abstract

**Background:**

Untreated syphilis in pregnancy is associated with adverse clinical outcomes for the infant. Most syphilis infections occur in sub-Saharan Africa (SSA), where coverage of antenatal screening for syphilis is inadequate. Recently introduced point-of-care syphilis tests have high accuracy and demonstrate potential to increase coverage of antenatal screening. However, country-specific cost-effectiveness data for these tests are limited. The objective of this analysis was to evaluate the cost-effectiveness and budget impact of antenatal syphilis screening for 43 countries in SSA and estimate the impact of universal screening on stillbirths, neonatal deaths, congenital syphilis, and disability-adjusted life years (DALYs) averted.

**Methods and Findings:**

The decision analytic model reflected the perspective of the national health care system and was based on the sensitivity (86%) and specificity (99%) reported for the immunochromatographic strip (ICS) test. Clinical outcomes of infants born to syphilis-infected mothers on the end points of stillbirth, neonatal death, and congenital syphilis were obtained from published sources. Treatment was assumed to consist of three injections of benzathine penicillin. Country-specific inputs included the antenatal prevalence of syphilis, annual number of live births, proportion of women with at least one antenatal care visit, per capita gross national income, and estimated hourly nurse wages. In all 43 sub-Saharan African countries analyzed, syphilis screening is highly cost-effective, with an average cost/DALY averted of US$11 (range: US$2–US$48). Screening remains highly cost-effective even if the average prevalence falls from the current rate of 3.1% (range: 0.6%–14.0%) to 0.038% (range: 0.002%–0.113%). Universal antenatal screening of pregnant women in clinics may reduce the annual number of stillbirths by up to 64,000, neonatal deaths by up to 25,000, and annual incidence of congenital syphilis by up to 32,000, and avert up to 2.6 million DALYs at an estimated annual direct medical cost of US$20.8 million.

**Conclusions:**

Use of ICS tests for antenatal syphilis screening is highly cost-effective in SSA. Substantial reduction in DALYs can be achieved at a relatively modest budget impact. In SSA, antenatal programs should expand access to syphilis screening using the ICS test.

*Please see later in the article for the Editors' Summary*

## Abstract 2

Yukari Manabe and colleagues evaluate the cost-effectiveness and budget impact of antenatal syphilis screening for 43 countries in sub-Saharan Africa and estimate the impact of universal screening on averted stillbirths, neonatal deaths, congenital syphilis, and DALYs.

*Please see later in the article for the Editors' Summary*

## Editors' Summary

**Background:**

Syphilis is a sexually transmitted infection caused by a bacterium called *Treponema pallidum*. In many countries, the screening and treatment program for syphilis in pregnancy is inadequate, leading to babies being affected. It is estimated that between 2.5% and 17% of pregnant women in sub-Saharan Africa are infected with syphilis; recent estimates suggest that more than 535,000 pregnancies occur in women with active syphilis each year. Maternal syphilis in pregnancy has been estimated to cause approximately half a million adverse outcomes in babies, including stillbirths, neonatal deaths, preterm or low-birth-weight babies, and congenital infections. If a pregnant woman is tested, and given penicillin if positive, then many of these harmful outcomes can be avoided. The best time to screen and treat is in the first half of pregnancy.

Until recently, tests for syphilis were done in a laboratory as an enzyme immunoassay, requiring technical staff. Recently, rapid tests for syphilis became available for use at the point of care. There are considerable advantages to this approach: the tests can be undertaken using a finger prick to obtain a small amount of blood, and require minimal staff training and no specialist laboratory equipment. A result can be given within minutes, avoiding the need for a return visit in settings where antenatal care is infrequent.

**Why Was This Study Done?:**

Although the advantages to the mother and baby of testing seem clear, the cost-effectiveness of a screening and treatment program using rapid point-of-care tests has not previously been assessed for most sub-Saharan African countries. The program has the greatest potential value in settings where syphilis is common. In local guidelines, testing is often recommended, but uncertainty over the costs and technical requirements has meant that antenatal syphilis screening has not been comprehensively introduced. The aim of this study was to assess whether antenatal syphilis screening was cost-effective for 43 countries in sub-Saharan Africa by estimating the extent of infant mortality and disability that could be prevented if maternal syphilis was diagnosed and treated.

**What Did the Researchers Do and Find?:**

The researchers created a model that allowed them to determine the cost-effectiveness of antenatal syphilis screening and treatment. They included many factors including the performance of the test, how common syphilis is in each country, the number of births, the likelihood of harmful outcomes, the effectiveness of penicillin therapy, the cost of an antenatal visit, and the cost of the test and the penicillin treatment, if positive. Then they calculated how many deaths and how much disability could be prevented by screening and treatment. The results were expressed in disability-adjusted life years (DALYs), which give the number of years affected by ill health, disability, or early death. The study found that screening was highly cost-effective, with each DALY prevented on average costing only US$11.

**What Do These Findings Mean?:**

Across sub-Saharan Africa, only about 40% of women are screened for syphilis during one of their antenatal care visits. These findings suggest that it would be an efficient use of health care resources to scale up antenatal screening programs for syphilis using the rapid point-of-care test. Comparing these results to those for other health care interventions in resource-limited settings suggests that screening pregnant women for syphilis in sub-Saharan Africa could achieve a substantial improvement in public health at relatively little cost. The researchers propose that combining HIV and syphilis tests into one antenatal screening package could be an efficient way of introducing a care package into settings where uptake is currently limited.

**Additional Information:**

Please access these websites via the online version of this summary at http://dx.doi.org/10.1371/journal.pmed.1001545.

- The World Health Organization provides information on syphilis in pregnant women
- The London School of Hygiene & Tropical Medicine has information on efforts to reduce congenital syphilis and a rapid syphilis test toolkit
- The US Centers for Disease Control and Prevention's STD Curriculum includes materials on syphilis

## Introduction

Syphilis infection is an important public health problem and causes significant perinatal morbidity and mortality in sub-Saharan Africa (SSA) [1],[2]. It is estimated that between 2.5% and 17% of pregnant women in SSA are infected with syphilis; recent estimates suggest that more than 535,000 pregnancies occur in women with active syphilis each year [3],[4]. Syphilis is a sexually transmitted disease caused by the spirochete *Treponema pallidum* and is of particular concern in pregnancy because of the risk of transmission to the fetus. Maternal syphilis confers an extremely high risk of adverse pregnancy outcomes: in a recent meta-analysis, 53.4%–81.8% of women with untreated syphilis had adverse outcomes, compared to 10.2%–20.8% of women without syphilis [5]. Neonates who survive with congenital syphilis are at risk for a range of severe effects, including low birth weight, premature delivery, congenital anomalies, active syphilis in the infant, and longer-term sequelae, which include deafness and neurologic impairment [6],[7]. These consequences are avoidable if infected mothers are identified before the third trimester of pregnancy and treated at least 30 d before delivery with three intramuscular doses of benzathine penicillin [8]–[10]. Although three doses are recommended in developed countries, data from Tanzania showed no increased risk for adverse pregnancy outcomes for seropositive women who received only one dose of benzathine penicillin compared to those who were seronegative [11]. A meta-analysis of interventional studies showed a decrease in stillbirths, perinatal deaths, and incidence of congenital syphilis with testing and treatment of syphilis in pregnant women.

Nontreponemal and treponemal serologic tests are used to diagnose *T. pallidum* [8]–[10]. Previously, treponemal tests were available only in enzyme immunoassay formats and were used as confirmatory tests. More recently, immunochromatographic strip (ICS) tests that detect serum antibodies to recombinant *T. pallidum*–specific antigens are one of the new options for syphilis screening among pregnant women attending antenatal clinics in Africa [12],[13]. This test has been found to be reliable and rapid, does not require refrigeration or specialized equipment, and can be performed in non-laboratory settings in only eight minutes to guide clinical decision making [13]. A recent meta-analysis showed that rapid point-of-care treponemal tests have sensitivity and specificity estimates comparable to those of lab-based treponemal tests [14]. In high prevalence settings such as SSA, such tests can be used as a screening tool. We sought to examine the cost-effectiveness of using point-of-care ICS tests to screen for syphilis in 43 countries in SSA where data on syphilis prevalence were available. In these settings, syphilis testing is often recommended, but has not been previously implemented due to cost and technical requirements.

## Methods

### Overview

**Figure 1:**
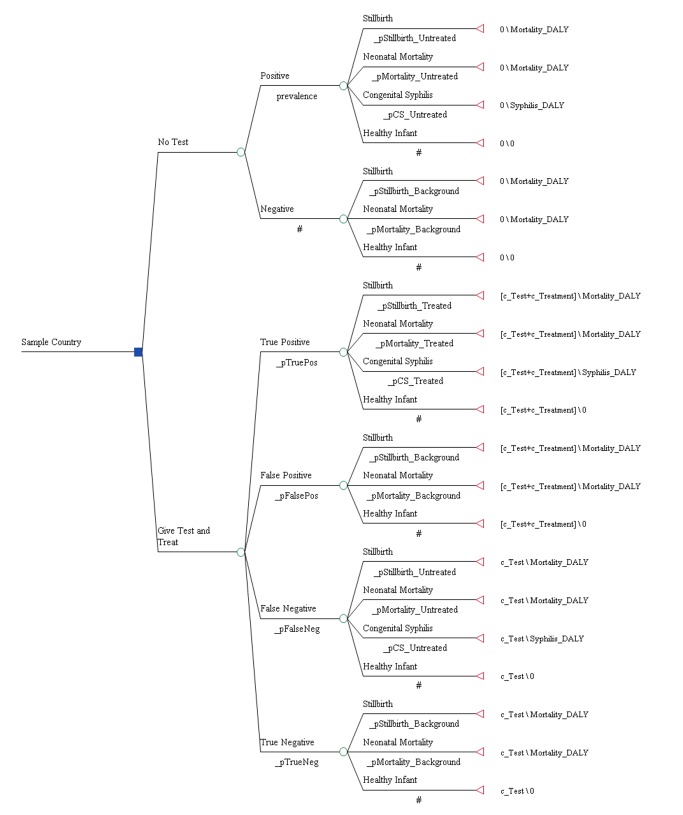
Decision tree.

The analysis was based on a decision analytic model (see Figure 1) that evaluated the cost-effectiveness of ICS testing and subsequent treatment with three injections of benzathine penicillin relative to no testing and no treatment in the setting of national antenatal care programs and from the perspective of the national health care system. Direct or indirect patient costs such as costs associated with travel to and from the health care facility were not included. The clinical benefit of treatment in the model was restricted to the infant and applied to a reduction in the probability of stillbirth, early neonatal death, as well as congenital syphilis. A separate decision tree was developed for each of the 43 countries in this analysis.

Table 1 displays the model parameters that are applied uniformly across all countries included in the model. The diagnostic accuracy of the ICS test was based on a recently published systematic review of the test's sensitivity and specificity under field conditions in antenatal care clinics in resource-poor settings [15]. Using the reported accuracy of the ICS test and local prevalence estimates for syphilis, we estimated the total expected number of true positive (prevalence×sensitivity of 86%), false positive ([1−prevalence]×[1−specificity of 99%]), true negative ([1−prevalence]×specificity of 99%), and false negative (prevalence×[1−sensitivity of 86%]) test results. Each of these four outcomes was then assigned a probability that one of the three clinical events (stillbirth, neonatal death, or congenital syphilis) in the model would occur [5]. In the comparator arm of the model, the two outcomes included were true negative (1 – prevalence) and untreated true positive (prevalence) syphilis cases and were similarly assigned an event probability [5]. For each country, the model then translated these three outcomes into disability-adjusted life years (DALYs). The years of life lost due to stillbirth and neonatal death were calculated by discounting the average reported local life expectancy at birth [16] using a discount rate of 3% [17]. The years of life lived with disability were calculated by applying an established disability weight for congenital syphilis of 0.315 [18] to the average life expectancy at birth and also discounted at a rate of 3%.

**Table 1:** General model inputs.

Model Input	Base Case	95% CI		Distribution	Reference
ICS test sensitivity	86.0%	74.5%	94.1%	Beta	Tucker et al. [15]
ICS test specificity	99.0%	97.8%	99.7%	Beta	Tucker et al. [15]
Stillbirth, mother with untreated syphilis	25.6%	17.8%	33.4%	Beta	Gomez et al. [5]
Neonatal mortality, mother with untreated syphilis	12.3%	9.1%	15.9%	Beta	Gomez et al. [5]
Congenital syphilis, mother with untreated syphilis	15.5%	7.1%	26.2%	Beta	Gomez et al. [5]
Stillbirth, mother without syphilis	4.6%	2.9%	6.8%	Beta	Gomez et al. [5]
Penicillin effectiveness in reducing stillbirths, RR	42.0%	16.1%	91.0%	Log-normal	Hawkes et al. [25]
Penicillin effectiveness in reducing neonatal mortality, RR	46.0%	25.0%	79.0%	Log-normal	Hawkes et al. [25]
Penicillin effectiveness in reducing congenital syphilis, RR	33.5%	18.0%	57.0%	Log-normal	Hawkes et al. [25] a
Congenital syphilis disability weight	0.315	0.159	0.471	Normal	Murray and Lopez [18]
Discount rate	3.0%	1.4%	5.3%	Beta	WHO [17] b

a Reference [25] does not report a 95% CI for this end point. We approximated the 95% CI by calculating the weighted average of the lower and upper confidence ranges for the three last studies reported in Figure 4A of [25] (page 689).

b Reference [17] suggests a range of 0%–6% for one-way sensitivity analyses, but does not report a suggested 95% confidence interval for PSAs.

RR, relative risk.

In order to estimate the net incremental impact of universal syphilis screening in the antenatal care setting on costs and outcomes, each model was first populated with the total number of reported live births in the country [16], which was then weighted by the observed proportion of women accessing at least one antenatal care visit through existing health delivery systems [16]. We then populated the comparator arm of the model with data on the percentage of women that are currently reported to be screened for syphilis in the antenatal care setting [19] and assumed that the baseline screening rate increased from the reported rate to an aspirational target of 100%. Several field studies have approached this rate [20],[21]. The baseline screening rate is available for 23 of the 43 countries we modeled. For the remaining 20 countries, we assumed a baseline screening rate equivalent to the reported weighted average for the 23 countries for which data are available (40.7%), which is in line with other reports of antenatal syphilis screening rates across SSA of 38% [6].

The total incremental cost of screening was calculated by multiplying the estimated absolute increase in the number of screened women in each country by the cost of the ICS test, plus the cost of the nursing time required to administer the test. The cost of antibiotic therapy for women testing positive for syphilis was defined as the cost of the drug and drug delivery equipment, plus the cost of the nursing time to administer the therapy. The alternative of no test and treatment carried a cost of zero. The incremental cost-effectiveness ratio (ICER) was calculated by dividing total cost by the difference in DALYs (e.g., cost/DALY averted). Select inputs were varied in one-way sensitivity analyses. In addition, probabilistic sensitivity analyses (PSAs) were performed by running 10,000 iterations of the model while randomly selecting the values for 14 key model inputs from a probability distribution that was defined for each of the parameters. This process enabled us to estimate the 95% confidence interval around the base case ICER estimates for each of the 43 countries included in the analysis, and it also allowed us to estimate the probability that the ICS test is cost-effective by calculating how many of the 10,000 iterations resulted in ICERs that fell below commonly accepted cost-effectiveness thresholds. Furthermore, we defined the prevalence target rate as the rate below which it would no longer be highly cost-effective to test for active syphilis in this setting, e.g., we reduced the prevalence rate in the model to such a level that the resulting ICER was equal to the cost-effectiveness threshold of one times per capita gross national income, as defined by the World Health Organization (WHO) [17],[22]. Finally, we compared that threshold rate to currently observed prevalence estimates. All calculations were performed in Excel 2007 (Microsoft) and TreeAge (TreeAge Software).

### Epidemiology and Health Outcomes

The prevalence of active syphilis in the antenatal care setting is reported in a number of epidemiological surveillance reports published by the WHO [19],[23],[24] as well as in studies published in the scientific literature (see Table 2). In the few cases where syphilis prevalence was not reported specifically for the antenatal care setting, we used prevalence from the general female population of child-bearing age (Cape Verde, Gabon, Gambia, and Zambia) or the general adult population (Uganda) instead. The most recent prevalence estimates varied from a minimum 0.6% in Senegal to a maximum of 14.0% in Equatorial Guinea. The population-weighted mean rate for SSA was 3.1%.

**Table 2:** Country-specific model inputs.

Country	Syphilis Prevalence [Reference] (Beta)	Percentage of Women Receiving Antenatal Syphilis Screening [19] a (Not Varied)	Number of Live Births [16] (Not Varied)	Antenatal Care Coverage [16] (Not Varied)	Neonatal Mortality Rate [26] (Beta)	Hourly Nurse Wage in US Dollars [43] (Log-Normal)
Angola	5.2% [23]	Not reported	795,000	80%	4.0%	$1.13
Benin	2.2% [24]	Not reported	350,000	84%	3.1%	$1.60
Botswana	0.9% [24]	Not reported	47,000	94%	3.7%	$5.74 [24],[44]
Burkina Faso	1.4% [19]	0.9%	713,000	85%	2.5%	$2.21
Burundi	3.2% [24]	Not reported	283,000	99%	2.8%	$0.84
Cameroon	2.3% [45]	Not reported	710,000	82%	3.2%	$6.19
Cape Verde^b^	1.9% [23]	Not reported	10,000	98%	0.8%	$2.57
Central African Republic	10.0% [19]	71.9%	154,000	69%	3.8%	$6.80
Chad	4.9% [23]	Not reported	503,000	53%	2.9%	$0.98 [46]
Comoros	4.6% [24]	0%	28,000	75%	2.2%	$1.99 [47]
Côte d'Ivoire	1.1% [48]	Not reported	673,000	85%	4.4%	$1.64
Democratic Republic of the Congo	3.3% [19]	2.1%	2,873,000	86%	3.5%	$0.31 [49]
Djibouti	3.1% [50]	63.3%	26,000	92%	2.9%	$5.42 [47]
Equatorial Guinea	14.0% [19]	35.8%	26,000	86%	3.0%	$3.21
Eritrea	1.7% [23]	0%	191,000	70%	1.9%	$0.79 [47]
Ethiopia	2.2% [19]	Not reported	2,613,000	28%	3.8%	$0.66
Gabon^c^	8.6% [51]	Not reported	41,000	94%	2.7%	$3.34
Gambia^d^	7.0% [23]	Not reported	66,000	98%	3.7%	$1.68
Ghana	3.6% [24]	9.0%	770,000	90%	2.6%	$0.97
Guinea	5.7% [23]	Not reported	390,000	88%	3.8%	$1.76
Guinea Bissau	1.1% [19]	Not reported	58,000	93%	3.6%	$1.23
Kenya	2.4% [52]	58.8%	1,529,000	92%	2.4%	$1.16
Lesotho	2.1% [24]	66.9%	60,000	92%	2.1%	$2.16
Liberia	13.6% [19]	10.9%	154,000	79%	4.8%	$0.68 [53]
Madagascar	5.6% [24]	84.7%	732,000	86%	2.4%	$0.41
Malawi	1.9% [24]	Not reported	663,000	92%	3.0%	$0.65
Mali	3.8% [24]	Not reported	714,000	70%	4.0%	$1.24
Mauritania	3.8% [24]	Not reported	117,000	75%	5.2%	$1.68
Mozambique	5.7% [19]	66.7%	883,000	92%	3.5%	$1.10
Namibia	1.7% [19]	93.8%	60,000	95%	2.1%	$5.71 [47]
Niger	1.5% [19]	Not reported	755,000	46%	2.6%	$2.50
Nigeria	3.0% [54]	Not reported	6,332,000	58%	4.0%	$2.55 [55]
Rwanda	1.5% [19]	75.2%	438,000	98%	3.5%	$2.00
Senegal	0.6% [56]	Not reported	465,000	87%	2.2%	$1.49
Sierra Leone	1.4% [19]	0%	226,000	87%	4.2%	$0.62 [57]
South Africa	1.9% [24]	74.5%	1,059,000	97%	1.5%	$8.25 [57]
Sudan	2.2% [19]	3.3%	1,429,000	64%	2.0%	$2.98 [47]
Swaziland	8.0% [24]	34.8%	35,000	97%	2.8%	$2.00 [58]
Tanzania	2.8% [19]	78.1%	1,862,000	88%	3.2%	$1.57 [59]
Togo	1.2% [19]	4.6%	193,000	87%	3.3%	$1.52
Uganda^e^	3.0% [24]	Not reported	1,514,000	94%	2.5%	$1.22 [47]
Zambia^f^	8.3% [24]	43.3%	600,000	94%	2.6%	$0.49
Zimbabwe	2.5% [60]	56.1%	374,000	90%	2.7%	$1.90

Distribution in the PSAs is given in parentheses in the column heads.

a The weighted average rate of 40.7% was used in cases where the proportion of women screened for syphilis infection in the antenatal care setting was not reported.

b The syphilis prevalence estimate of 1.9% was reported in the 2002 HIV/AIDS surveillance update by the WHO [23]. For this specific country, syphilis prevalence data from the antenatal care setting were not available, neither in one of the WHO reports nor in the scientific literature.

c The 2002 HIV/AIDS surveillance report by the WHO [23] reported a syphilis prevalence of 11% in the general female population; a study by Bertherat et al. [51] reported 8.6%, also in the general female population; and a joint report by the WHO, Joint United Nations Programme on HIV/AIDS, and United Nations Children's Fund [19] in the antenatal care setting reported 1%, although with a cautionary note that the data may not be nationally representative. Therefore, the lower of the two other estimates was used.

d The syphilis prevalence estimate of 7.0% was reported in the 2002 HIV/AIDS surveillance update by the WHO [23]. For this specific country, syphilis prevalence data from the antenatal care setting were not available, neither in one of the WHO reports nor in the scientific literature.

e The syphilis prevalence estimate of 3.0% was reported in the 2007 HIV/AIDS surveillance update by the WHO [24]. For this specific country, syphilis prevalence data from the antenatal care setting were not available, neither in one of the WHO reports nor in the scientific literature.

f The 2002 HIV/AIDS surveillance report by the WHO [23] reported a syphilis prevalence of 20.3% in the antenatal care setting, the 2007 HIV/AIDS surveillance report by the WHO [24] reported 8.3% in the general female population, and a joint report by the WHO, Joint United Nations Programme on HIV/AIDS, and United Nations Children's Fund [19] in the antenatal care setting reported 5.3%, although with a cautionary note that the data may not be nationally representative. Therefore, the lower of the two other estimates was used.

For seropositive untreated women, the rates of stillbirth, early neonatal death, and congenital syphilis have been previously reported at 25.6%, 12.3%, and 15.5%, respectively [5]. We applied these three estimates to seropositive women in the no screen and test arm, as well as to women with a false negative test result in the screen and test arm for all countries in our model. A recently published meta-analysis of the effectiveness of antibiotic therapy in the treatment of syphilis infection among pregnant women in resource-limited settings estimated the average risk of stillbirth, neonatal death, and congenital syphilis to decrease by 58%, 54%, and 66.5%, respectively, in women receiving at least one dose of penicillin [25]. Thus, we assumed that the risk of stillbirth would decrease to 10.8% (e.g., 25.6% reduced by 58%), the risk of neonatal death to 5.7% (e.g., 12.3% reduced by 54%), and the risk of congenital syphilis to 5.2% (e.g., 15.5% reduced by 66.5%) in women with a true positive ICS test result who subsequently received penicillin therapy. For the true negative and false positive end points in our model, we assumed a risk of congenital syphilis of 0%, a risk of stillbirth of 4.6% [5], and a country-specific early neonatal mortality rate that varied from 0.8% in Cape Verde to 5.2% in Mauritania (see Table 2) [26].

### Direct Medical Costs

Direct medical costs were expressed in 2011 US dollars and consisted of several components—the cost of the ICS test plus the human resource cost to administer it and interpret the results applied to all patients. For patients with a positive test result, there were the additional costs of one benzathine penicillin injection immediately following the positive result, two additional injections at follow-up visits, and the human resource cost of three penicillin administrations. Previous cost-effectiveness studies reported the cost per ICS test, not including administration, to be in the range of US$0.74–US$1.83. Yet these studies employed cost data from 2003–2004, and since the ICS test had just come to market at that time, prices may have subsequently declined. A recent internet search of the terms “treponemal test kit” showed that these tests currently retail for US$0.20–US$0.50 per unit [27]. However, in order to account for additional equipment that may be required over and above what is contained in a test kit (e.g., gloves), we used the low end of the historical range in our model (e.g., US$0.74) [28]. Similarly, the cost of a single dose of benzathine penicillin injection, excluding the cost of administration, has been reported to be in the range of US$0.77–US$1.92, and we used the higher estimate of that range (US$1.92) [7]. Both the cost per test and the cost per treatment were assumed to be identical across the 43 countries in the model. Furthermore, we assumed that infants born with congenital syphilis would not produce an increase in direct medical costs over their lifetimes.

The human resource costs were calculated assuming that each test was performed in the context of a nurse visit that lasted an average of 17.5 min [29], while each positive test resulted in an additional visit immediately following the positive result and two follow-up visits, which each lasted an average of 20 min [29]. For 28 countries in the model, hourly nurse salaries were estimated on the basis of a recently published analysis that used data from the Occupational Wages around the World database to estimate current nurse wages. Wage information for the remaining 15 countries was based on other databases or on estimates reported in the scientific literature (see Table 2). Since all relevant costs were accrued at the point of care and no future cost offsets were assumed, it was not necessary to apply a discount rate to our cost estimates.

## Results

Model results are displayed in Table 3. The annual number of live births in SSA is reported at almost 32 million, of which approximately 23.5 million (e.g., 74%) were from women reported to have had at least one antenatal care visit. The greatest number of infants is born in the most populous African country of Nigeria, with over 6 million live births, although a relatively low proportion of Nigerian women (reported at 58%) receive antenatal care and are included in our analysis. The estimated average cost per syphilis screening ranged from US$0.83 to US$3.15, with a median cost of US$1.22. The cost of the full treatment course was estimated to range from US$6.07 to US$14.01, with a median cost of US$7.40. In sum, the incremental direct medical cost associated with increasing screening rates from current levels to universal screening for all 23.5 million women that receive antenatal care in SSA is estimated at US$20.8 million per year, with a breakdown of the budget impact per country reported in Table 3.

**Table 3:** Model results.

Country	Potential Stillbirths Averted	Potential Neonatal Deaths Averted	Potential Cases of Congenital Syphilis Averted	Potential DALYs Averted	Potential Increase in Direct Medical Cost in US Dollars	Cost/DALY Averted in US Dollars (95% CI)	Probability Screening Is Cost-Effective	Prevalence Target Rate	Current/Target Prevalence Rate
Angola	2,912^a^	1,120^a^	1,466^a^	117,366^a^	$544,320^a^	$5 ($3–$13)	99.8%	0.005%	1,040
Benin	569^a^	219^a^	287^a^	23,837^a^	$247,137^a^	$10 ($5–$45)	99.8%	0.029%	76
Botswana	35^a^	13^a^	18^a^	1,434^a^	$68,566^a^	$48 ($15–$925)	99.6%	0.006%	150
Burkina Faso	1,248	480	629	51,892	$936,644	$18 ($6–$189)	99.4%	0.045%	31
Burundi	789^a^	304^a^	398^a^	31,549^a^	$204,683^a^	$6 ($3–$20)	99.8%	0.077%	42
Cameroon	1,179^a^	454^a^	594^a^	47,521^a^	$1,000,854^a^	$21 ($9–$90)	99.8%	0.040%	58
Cape Verde	16	6	8	752	$9,919	$13 ($5–$74)	99.8%	0.007%	271
Central African Republic	443	171	223	17,404	$116,893	$7 ($4–$18)	99.8%	0.113%	88
Chad	1,150^a^	442^a^	579^a^	45,566^a^	$217,206^a^	$5 ($3–$13)	99.8%	0.029%	169
Comoros	143	55	72	6,195	$35,720	$6 ($3–$17)	99.8%	0.031%	148
Côte d'Ivoire	554^a^	213^a^	279^a^	23,029^a^	$462,013^a^	$20 ($6–$551)	98.6%	0.021%	52
Democratic Republic of the Congo	11,852	4,560	5,969	465,270	$2,567,366	$6 ($3–$17)	99.7%	0.088%	38
Djibouti	40	16	20	1,714	$23,941	$14 ($7–$46)	99.9%	0.033%	94
Equatorial Guinea	298	115	150	12,027	$40,681	$3 ($2–$9)	99.8%	0.002%	7,000
Eritrea	337	130	170	14,577	$151,156	$10 ($4–$67)	99.7%	0.041%	41
Ethiopia	1,417^a^	545^a^	714^a^	60,491^a^	$484,906^a^	$8 ($4–$33)	99.7%	0.043%	51
Gabon	292^a^	112^a^	147^a^	12,676^a^	$56,440^a^	$4 ($2–$12)	99.8%	0.004%	2,150
Gambia	399^a^	153^a^	201^a^	16,909^a^	$66,944^a^	$4 ($2–$10)	99.9%	0.037%	189
Ghana	3,371	1,297	1,698	147,991	$817,586	$6 ($3–$17)	99.9%	0.013%	277
Guinea	1,722^a^	663^a^	867^a^	71,075^a^	$344,190^a^	$5 ($3–$13)	99.8%	0.053%	108
Guinea Bissau	52^a^	20^a^	26^a^	2,051^a^	$39,509^a^	$19 ($6–$524)	97.7%	0.036%	31
Kenya	2,048	788	1,031	86,304	$725,991	$9 ($4–$35)	99.7%	0.024%	75
Lesotho	57	22	29	2,236	$29,036	$13 ($6–$59)	99.8%	0.022%	95
Liberia	2,189	842	1,102	91,622	$189,395	$2 ($1–$5)	99.8%	0.073%	186
Madagascar	801	308	403	35,512	$117,182	$3 ($2–$9)	99.7%	0.035%	160
Malawi	1,020^a^	393^a^	514^a^	42,106^a^	$396,479^a^	$9 ($4–$49)	99.7%	0.052%	37
Mali	1,672^a^	643^a^	842^a^	67,400^a^	$414,013^a^	$6 ($3–$17)	99.8%	0.035%	109
Mauritania	294^a^	113^a^	148^a^	12,453^a^	$80,413^a^	$6 ($4–$19)	99.8%	0.022%	173
Mozambique	2,289^a^	881^a^	1,153^a^	91,501^a^	$395,125^a^	$4 ($3–$11)	99.7%	0.044%	130
Namibia	9	3	4	387	$9,492	$24 ($9–$174)	99.8%	0.009%	189
Niger	459^a^	176^a^	231^a^	18,927^a^	$341,028^a^	$18 ($7–$152)	99.5%	0.077%	19
Nigeria	9,604	3,695	4,837	387,087	$3,869,208	$10 ($5–$31)	99.8%	0.024%	124
Rwanda	237	91	119	9,855	$159,799	$16 ($6–$147)	99.6%	0.043%	35
Senegal	196^a^	75^a^	99^a^	8,362^a^	$307,168^a^	$37 ($11–$949)	97.6%	0.020%	28
Sierra Leone	409	157	206	15,893	$208,526	$13 ($5–$136)	99.3%	0.055%	25
South Africa	739	284	372	30,028	$920,106	$31 ($12–$170)	99.8%	0.008%	238
Sudan	2,889^a^	1,111^a^	1,455^a^	124,777^a^	$1,644,950^a^	$13 ($6–$58)	99.8%	0.022%	100
Swaziland	263	101	132	10,322	$44,491	$4 ($3–$11)	99.8%	0.008%	1,000
Tanzania	1,492	574	751	62,868	$518,771	$8 ($4–$28)	99.7%	0.041%	68
Togo	285	110	144	12,027	$212,966	$18 ($6–$310)	98.8%	0.039%	31
Uganda	3,759^a^	1,446^a^	1,893^a^	155,120^a^	$1,133,929^a^	$7 ($4–$23)	99.8%	0.041%	73
Zambia	3,941	1,516	1,985	156,128	$442,829	$3 ($2–$7)	99.8%	0.015%	553
Zimbabwe	549	211	276	21,922	$226,479	$11 ($5–$38)	99.8%	0.039%	64
Sum/weighted average	64,023	24,630	32,242	2,614,162	$20,824,049	$11 ($5–$77)	99.7%	0.038%	82

a We assumed that the proportion of women tested for syphilis in the antenatal care setting in the 20 African countries that do not report such data is equal to the weighted average (40.7%) of the 23 African countries that do report it. Should the current rate of syphilis screening turn out to be lower than 40.7% in these cases, the number of events and DALYs averted, as well as direct medical cost, would increase proportionately.

The base case results of the model suggest that universal access to syphilis screening, relative to current rates, may reduce the incidence of stillbirth in SSA by up to 64,000 cases annually, the incidence of neonatal death by up to 25,000 cases, and the incidence of congenital syphilis by up to 32,000 cases, and result in an annual reduction of up to 2.6 million DALYs. Because of the size of their populations, the largest impact on outcomes would be expected to occur in Nigeria and Democratic Republic of the Congo, with a potential annual reduction in DALYs of up to 387,000 and 465,000, respectively. In countries where current syphilis screening rates are reported to be relatively high, such as Namibia at 93.8%, the incremental benefit of universal screening is expected to be more modest, at up to 387 DALYs averted.

**Figure 2:**
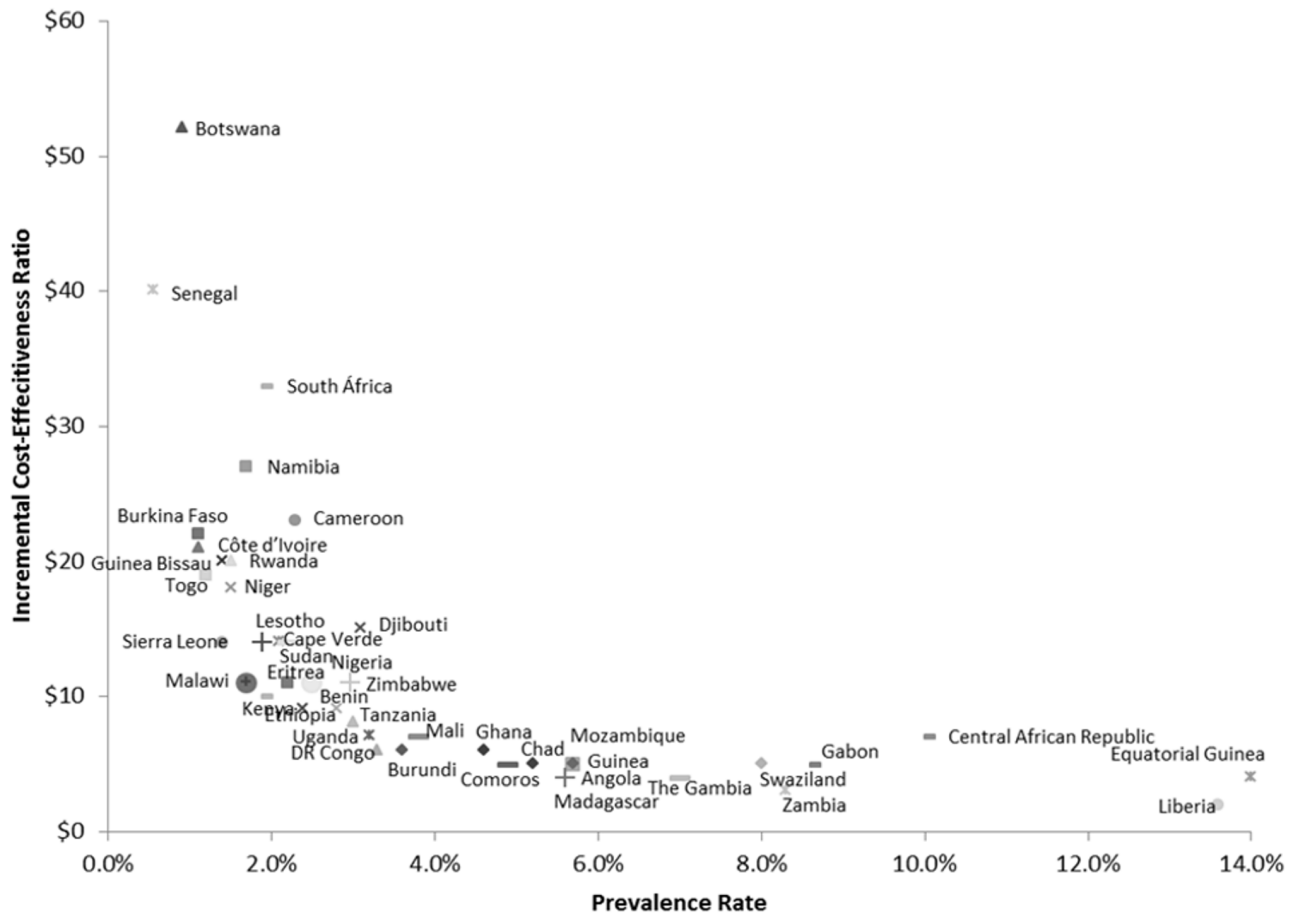
Country ICERs by prevalence. DR Congo, Democratic Republic of the Congo.

The cost-effectiveness of antenatal syphilis screening for 43 countries in SSA is reported in Table 3 and Figure 2. We estimate the ICERs to range from a low of US$2 (95% CI: US$1–US$5) in Liberia to a high of US$48 (95% CI: US$15–US$925) in Botswana. Out of the 43 countries in the model, we estimate a cost per DALY averted of less than US$20 in 37 cases, and less than US$10 in 23 cases. The population-weighted average ICER for all 43 sub-Saharan African countries studied is estimated at US$11 per DALY averted.

### Uncertainty Analyses

Select model inputs were varied in one-way sensitivity analyses (Table 4). For simplicity, we report only the high end of the sensitivity range. In the base case, the mean ICER was US$11 (range: US$2–US$48). All model results proved robust to a 50% relative decrease in syphilis prevalence (range: US$3–US$94), a decrease in the sensitivity of the ICS test to from 86% to 75% (range: US$2–US$50), a decrease in the specificity of the ICS test from 99% to 91% (range: US$3–US$65), a 25% relative increase in nurse wages (range: US$2–US$56), a 25% increase in the cost of the ICS test kit and penicillin injection (range: US$4–US$72), a reduction in the effectiveness of penicillin treatment in reducing the probability of stillbirth from 58% to 7% (range: US$5–US$111), a decrease in the neonatal death rate from 54% to 18% (range: US$3–US$57), a decrease in the rate of congenital syphilis from 75% to 43% (range: US$2–US$50), and an increase in the discount rate from 3% to 6% (range: US$3–US$79).

**Table 4:** One-way sensitivity analyses—cost per DALY averted.

Country	Syphilis Prevalence Decreased 50%^a^	ICS Sensitivity 75%^b^	ICS Specificity 91%^b^	Nurse Wages Increased 25%^a^	Equipment $2/ICS Test and $3/Penicillin Administration^a^	Penicillin Efficacy in Reducing Stillbirth, RR = 0.93^b^	Penicillin Efficacy in Reducing Neonatal Death, RR = 0.82^b^	Penicillin Efficacy in Reducing Congenital Syphilis, RR = 0.57^b^	Discount Rate 6%^c^
Angola	$8	$5	$6	$5	$9	$11	$6	$5	$8
Benin	$20	$11	$15	$11	$20	$24	$12	$11	$17
Botswana	$94	$50	$65	$56	$72	$111	$57	$50	$79
Burkina Faso	$35	$19	$25	$20	$33	$42	$22	$19	$30
Burundi	$12	$7	$9	$7	$14	$15	$8	$7	$11
Cameroon	$40	$22	$28	$25	$31	$49	$25	$22	$35
Cape Verde	$25	$14	$18	$15	$24	$31	$16	$14	$24
Central African Republic	$12	$7	$8	$8	$9	$16	$8	$7	$11
Chad	$9	$5	$7	$5	$10	$11	$6	$5	$8
Comoros	$11	$6	$8	$6	$11	$13	$7	$6	$10
Côte d'Ivoire	$39	$21	$29	$22	$40	$47	$24	$21	$34
Democratic Republic of the Congo	$10	$6	$8	$6	$13	$13	$7	$6	$9
Djibouti	$26	$14	$18	$16	$21	$32	$17	$15	$24
Equatorial Guinea	$5	$3	$4	$4	$5	$8	$4	$4	$6
Eritrea	$20	$11	$15	$11	$23	$24	$12	$11	$18
Ethiopia	$15	$8	$12	$8	$18	$19	$10	$8	$14
Gabon	$8	$4	$6	$5	$7	$10	$5	$5	$8
Gambia	$7	$4	$5	$4	$7	$9	$5	$4	$7
Ghana	$10	$6	$8	$6	$11	$13	$7	$6	$10
Guinea	$9	$5	$6	$5	$9	$11	$6	$5	$8
Guinea Bissau	$38	$20	$28	$21	$40	$45	$23	$20	$31
Kenya	$21	$9	$12	$9	$18	$20	$10	$9	$15
Lesotho	$25	$13	$18	$14	$24	$30	$16	$14	$21
Liberia	$3	$2	$3	$2	$4	$5	$2	$2	$3
Madagascar	$6	$3	$5	$3	$7	$8	$4	$3	$6
Malawi	$18	$10	$14	$10	$21	$22	$11	$10	$16
Mali	$11	$6	$9	$7	$12	$14	$7	$6	$10
Mauritania	$12	$7	$9	$7	$12	$15	$8	$7	$11
Mozambique	$8	$4	$6	$5	$9	$10	$5	$5	$7
Namibia	$47	$25	$33	$29	$37	$57	$29	$26	$42
Niger	$35	$19	$25	$20	$33	$42	$22	$19	$30
Nigeria	$19	$10	$14	$11	$18	$23	$12	$10	$16
Rwanda	$31	$17	$23	$18	$31	$38	$19	$17	$27
Senegal	$72	$38	$53	$40	$74	$85	$44	$38	$63
Sierra Leone	$25	$14	$19	$14	$30	$30	$16	$14	$21
South Africa	$59	$32	$40	$36	$42	$71	$37	$32	$51
Sudan	$25	$14	$18	$15	$23	$31	$16	$14	$23
Swaziland	$7	$4	$6	$5	$8	$10	$5	$4	$7
Tanzania	$16	$9	$12	$9	$16	$19	$10	$9	$14
Togo	$34	$18	$25	$19	$35	$41	$21	$18	$30
Uganda	$14	$8	$10	$8	$15	$17	$9	$8	$12
Zambia	$5	$3	$4	$3	$6	$7	$3	$3	$5
Zimbabwe	$20	$11	$14	$11	$20	$24	$12	$11	$17

All costs in US dollars. Incremental cost-effectiveness thresholds obtained in one-way sensitivity analyses were calculated by replacing the base case estimates of a single parameter in the model with a very high and a very low value, while leaving all other model parameters unchanged. For space considerations, Table 4 reports only the upper end of the new ICER range.

a No 95% confidence interval was available for these parameters; we therefore used a subjective range for syphilis prevalence and nurse wages, and looked to cost estimates cited in other published sources to inform the sensitivity range for the ICS test and the cost of penicillin therapy.

b The sensitivity range was informed by 95% confidence intervals around the base case estimate.

c The sensitivity range of the discount rate followed WHO guidance [16].

RR, relative risk.

Furthermore, the PSAs suggest that the model results are also robust to simultaneous variation of all model parameters, as the 95% confidence intervals associated with the ICERs fall well below the relevant cost-effectiveness thresholds (Table 3). In addition, the number of iterations for the 43 countries that resulted in ICERs that would be considered to be highly cost-effective, as defined by the WHO [17],[22], generally exceeded 99% and was slightly below this rate only for four countries with relatively low syphilis prevalence: Côte d'Ivoire (98.6%), Guinea Bissau (97.7%), Senegal (97.6%), and Togo (98.8%).

As also displayed in Table 3, the results suggest that syphilis screening would remain highly cost-effective even at very low rates of syphilis prevalence, averaging 0.038% for the 43 sub-Saharan African countries studied, e.g., one positive case in roughly 2,630 pregnant women screened. In the relatively high-income countries of Angola, Equatorial Guinea, and Gabon, screening would remain highly cost-effective at prevalence rates below 0.005% (e.g., five positive cases out of 100,000 pregnancies).

Finally, we describe the relationship between currently observed syphilis prevalence rates and target rates—the rate below which it would no longer be highly cost-effective to test for active syphilis—in Table 3. For all 43 of the sub-Saharan African countries studied in aggregate, the current prevalence rate of 3.1% is 82 times higher than the target rate of 0.038%. However, there is considerable cross-country variation in this estimate. For example, the current rate is 19 times higher than the target rate in the case of Niger (1.5% versus 0.077%), whereas syphilis prevalence in Angola, Swaziland, Gabon, and Equatorial Guinea could fall by a factor of more than 1,000 and it would remain highly cost-effective to screen. In 21 out of the 43 countries modeled, current prevalence exceeds the target rate by a factor of 100 or more.

## Discussion

To our knowledge, this is the first study to evaluate the cost-effectiveness of antenatal syphilis screening across most sub-Saharan African countries. Using the commonly accepted threshold of an ICER less than one times per capita gross national income to define a highly cost-effective intervention would suggest that screening is highly cost-effective in all countries that were evaluated [17]. This finding is robust to variations of inputs in one-way sensitivity analyses. Furthermore, results from our PSAs suggest that the upper bounds of the 95% confidence intervals, as well as a very high proportion of iterations (generally exceeding 99%), result in ICERs that fall below these established thresholds. In addition, syphilis prevalence rates would have to fall dramatically before antenatal screening would stop yielding good value for health care resources.

Our base case results are largely in line with previous studies on the cost-effectiveness of antenatal syphilis screening using the plasma reagin test in resource-limited settings, which estimated a cost per DALY averted of US$4 in Zambia [30] (versus US$3 in our model; 95% CI: US$2–US$7), US$10 in Kenya [31],[32] (versus US$9 in our model; 95% CI: US$4–US$35), and US$11 in Tanzania [33] (versus US$8 in our model; 95% CI: US$4–US$28). All of these previously published estimates fall within the bounds of our 95% confidence intervals. More recent analyses that evaluated the cost-effectiveness of antenatal syphilis screening using the ICS test are available for Tanzania (ICER: US$12–US$17 [34]) and for SSA in general [7],[35]. The studies for SSA found that screening was cost-saving, e.g., it was associated with lower cost as well as improved outcomes.

Notwithstanding our assumption that every woman with a positive ICS test completes a full three-dose regimen of penicillin therapy, we believe that our methods are generally conservative as we bias our model against the intervention with respect to three relevant parameters. First, in our model, all clinical benefits of treatment apply only to the infant, although it is quite possible that additional benefits could accrue to either the mother herself through reductions in long-term mortality or morbidity associated with syphilis infection, or to the mother's sexual partners through a reduction in syphilis transmission. Second, antibiotic therapy may also reduce the incidence of other relevant clinical end points such as miscarriage, spontaneous abortion, or low birth weight, which are not included in our model. Third, unlike other studies published in the relevant literature [7],[35], we did not model lifetime treatment costs associated with congenital syphilis. Infants born with congenital syphilis may experience complications of the disease that can result in increased direct medical costs over their lifetime, in which case screening may partially offset these costs or even result in a net cost saving. The lifetime treatment costs for congenital syphilis were not modeled, because we could not be reasonably sure that these medical services would be routinely provided and paid for by the national health care systems across SSA.

Our results document a strong inverse relationship between syphilis prevalence and the cost-effectiveness of syphilis screening (Figure 2), i.e., the reduction in DALYs per health care dollar spent tends to be higher in countries with higher prevalence rates. This is primarily due to the fact that with increasing prevalence, the net health benefits increase at a faster rate relative to net costs, e.g., a doubling of the syphilis prevalence rate will double the DALYs averted as a result of screening and treatment, but the associated cost impact will be much less than double since the initial cost of screening is essentially fixed. Although we find that antenatal syphilis screening is highly cost-effective everywhere, this implies that countries with a relatively high prevalence rate exceeding the continental average of approximately 3% may constitute a particularly appealing target for intervention.

In the United Kingdom, antenatal syphilis screening is cost-effective at prevalence rates between 1 and 11 per 100,000 tested cases. At the current prevalence rate of about 5–6 per 100,000 tested cases, routine screenings are recommended by the National Institute for Health and Care Excellence, and British women are routinely screened for syphilis during pregnancy [36]. We estimate that with the availability of the relatively low-cost ICS test, the syphilis target rate may have fallen close to the rate observed in Britain, at least for the eight countries in SSA with relatively high per capita income (exceeding US$3,000 per year). In the two most extreme cases, it would remain highly cost-effective to screen for syphilis even if only one woman were to be infected out of the estimated 22,000 women that attend antenatal care each year in Equatorial Guinea, or two women out of the approximately 40,000 women attending antenatal care each year in Gabon. However, routine testing could be justified even among the six poorest countries with annual incomes of less than US$360 per year as long as the prevalence rates exceed 50–90 per 100,000 tested cases, which is substantially lower than current rates. In addition, given the relative simplicity of the ICS test, national payers across SSA, but especially in lower-income countries, should consider exploring task-shifting strategies that rely on specially trained non-nurse health workers to administer and interpret the ICS test to reduce its budget impact.

Worldwide, penicillin is the drug of choice for treating syphilis. Factors favoring its use include low cost, high efficacy in syphilis treatment, and decades of clinical experience with its use. However, the optimal regimen for treatment for syphilis in pregnancy has not been established in clinical trials. The US Centers for Disease Control and Prevention recommends that adults infected with syphilis within the previous 12 mo receive 2.4 million units of benzathine penicillin intramuscularly as a single dose, while adults with syphilis infection of unknown duration or of longer duration should be treated with 7.2 million units of benzathine penicillin administered as three doses of 2.4 million units each intramuscularly at 1-wk intervals [8],[10]. The rationale for additional doses is that the causative organism of syphilis divides more slowly in infections of longer duration; hence, more prolonged antibiotic treatment is needed to eliminate the infection.

Data are limited comparing the efficacy of one versus three doses of benzathine penicillin for syphilis treatment. For practical considerations related to implementation in resource-constrained settings, a previous cost-effectiveness analysis assumed treatment with a single dose of benzathine penicillin in SSA [7]. We applied the cost of a three-dose option in our model because we assumed that the majority of women screened will be unable to provide a date of onset of syphilis symptoms, and, consequently, these women should receive three doses based on current guidelines. Furthermore, there is some evidence in a setting known to have a high prevalence of HIV that the use of multiple doses of penicillin is associated with a reduced risk of pregnancy loss [37],[38].

A limitation of the model is that prevalence estimates may not be accurate for some countries. To address this, we calculated the prevalence target rate in the model. Also, the ICS test remains positive even after syphilis is treated, which may result in unnecessary treatment during subsequent pregnancies. This is important because fertility rates are high in most African countries. However, the risk of overtreatment is balanced by the fact that tracing and treatment of sexual contacts is often inadequate, and reinfection with syphilis is likely to occur in a subset of patients. Other limitations of the model include the use of estimated rather actual nurse wages in select countries. Furthermore, we assumed that there is no loss to follow-up and that all women receive all three penicillin injections, yet imperfect attendance to follow-up visits could reduce the effectiveness of penicillin therapy and, as a result, worsen the cost-effectiveness estimates. However, given that one dose of penicillin may also be sufficient to achieve the full effectiveness of penicillin therapy [11], it is not clear what impact imperfect attendance to follow-up visits may have on the cost-effectiveness estimates. In addition, penicillin hypersensitivity, which would require a different choice of antibiotic regimen, was not explicitly factored into our model. However, penicillin hypersensitivity is a fairly uncommon condition that may affect only about 1% of patients [39]. Lastly, we excluded the costs of training, outreach, and shipping of tests necessary to scale up antenatal syphilis testing across the continent, which may underestimate the true economic cost of making the ICS test more broadly available in the various local antenatal care settings.

Undoubtedly, additional resources will be required for implementation of antenatal syphilis screening using the ICS test in SSA. Nevertheless, our findings suggest that the budget impact associated with the health care resources required to provide universal syphilis screening in the antenatal care setting is relatively modest. For five countries, the budget impact may exceed US$1 million per year. However, these estimates ought to be viewed in the context of current levels of health-care-related spending in SSA. In a 2011 WHO report, only Tanzania had attained the African Union target of 15% of national budgets being spent on the health sector [40]. This suggests that additional domestic resources could be made available for health care interventions over most of the continent.

Furthermore, international funding targeted at improvement of maternal and child health services across the continent to attain the Millennium Development Goals could be used to support the scale-up of syphilis testing. Donor money targeted for infectious diseases such as the United States President's Emergency Plan for AIDS Relief is increasingly being recognized as an opportunity to strengthen health care systems to confront other diseases. One viable strategy involves integration of screening for syphilis via rapid test kits into an antenatal package that currently includes rapid HIV screening. Recently, countries in Latin America and Asia adopted an integrated approach to the prevention of mother-to-child transmission of HIV and syphilis, allowing for more efficient implementation of prevention services while minimizing the need for additional infrastructure and health care workers [41],[42]. We recommend a similar approach for sub-Saharan African countries.

## Supporting Information

Alternative Language Abstract S1 **Abstract translated into French by Mary Lou Bradley.**
(DOCX)

Alternative Language Abstract S2 **Abstract translated into Portuguese by Dr. Elsa Marques.**
(DOCX)

## Abbreviations

DALY: disability adjusted life year
ICER: incremental cost-effectiveness ratio
ICS: immunochromatographic strip
PSA: probabilistic sensitivity analysis
SSA: sub-Saharan Africa
WHO: World Health Organization
