# Supplementary material for: Antenatal Syphilis Screening Using Point-of-Care Testing in Sub-Saharan African Countries: A Cost-Effectiveness Analysis
Source: PLoS Med. 2013 Nov 5;10(11):e1001545. doi: 10.1371/journal.pmed.1001545 (PMC3818163; doi:10.1371/journal.pmed.1001545)
Supplement: Alternative Language Abstract S1 — Abstract translated into French by Mary Lou Bradley. (DOCX) [file pmed.1001545.s001.docx]

**Résumé**

**Contexte :** La syphilis non traitée pendant la grossesse est associée à des résultats cliniques indésirables pour le nourrisson. La plupart des infections de la syphilis se produisent en Afrique subsaharienne où la couverture du dépistage prénatal de la syphilis est insuffisante. Des tests de dépistage de la syphilis point de service récemment introduits sont de haute précision et démontrent le potentiel d’élargir l’accès de la couverture du dépistage prénatal. Pourtant, les données du rapport coût-efficacité spécifiques par pays sont très limitées. L’objectif de la présente analyse était d’évaluer le rapport coût-efficacité et l’impact budgétaire du dépistage de la syphilis prénatale pour 43 pays en Afrique subsaharienne et l’impact estimé du dépistage universel sur les mortinaissances, les décès néonatals, la syphilis congénitale et les années de vie corrigées de l’incapacité (AVCI) évitées.

**Méthodes et Résultats:** Le modèle de décision analytique reflète la perspective du système nationale de la santé et il est basé sur la sensibilité (86%) et la spécificité (99%) rapportées pour les tests sur bandelettes immunochromatographiques (BIC). Les résultats cliniques pour les enfants nés de mères infectées par syphilis sur les points d’aboutissement de mortinaissance, de décès néonatal et de syphilis congénitale ont été procurés de sources publiées. Le traitement a été basé sur l’hypothèse de trois injections de benzathine-pénicilline. Les données d’entrée spécifiques par pays ont compris la prévalence prénatale de la syphilis; le nombre annuel de naissances vivantes; la proportion de femmes ayant eu au moins une visite prénatale; le revenue national brut par habitant; et l’estimation du salaire horaire d’une infirmière. Dans tous les 43 pays de l’Afrique subsaharienne, le dépistage de la syphilis a un rapport coût-efficacité élevé avec un coût moyen/AVCI évitées de US$11 (étendue: US$2 – US$48). Le rapport coût-efficacité du dépistage reste élevé même si la prévalence moyenne baisse de la fréquence actuelle de 3,1% (étendue: 0,6%-14,0%) à 0,038% (étendue: 0,002% - 0,113%). Le dépistage prénatal universel des femmes enceintes effectué dans une clinique pourrait réduire le nombre annuel de mortinaissances jusqu’à 64 000; de décès néonataux jusqu’à 25 000; la fréquence annuelle de la syphilis congénitale jusqu’à 32 000; et pourrait éviter jusqu’à 2,6 millions AVCI pour un coût médical direct estimé à US$20,8 millions par an.

**Conclusions:** L’utilisation des tests sur BIC pour le dépistage de la syphilis prénatale a un rapport coût-efficacité très haut en Afrique subsaharienne. Une réduction importante des AVCI peut être réalisée avec un impact budgétaire relativement modeste. En Afrique subsaharienne, les programmes prénatals devraient élargir l’accès au dépistage de la syphilis en utilisant le test sur BIC.
