# Supplementary material for: Antenatal Syphilis Screening Using Point-of-Care Testing in Sub-Saharan African Countries: A Cost-Effectiveness Analysis
Source: PLoS Med. 2013 Nov 5;10(11):e1001545. doi: 10.1371/journal.pmed.1001545 (PMC3818163; doi:10.1371/journal.pmed.1001545)
Supplement: Alternative Language Abstract S2 — Abstract translated into Portuguese by Dr. Elsa Marques. (DOCX) [file pmed.1001545.s002.docx]

**Resumo**

**Enquadramento:** A sífilis não tratada durante a gravidez é associada a resultados clínicos adversos para a criança. A maiorida das infecções por sífilis ocorrem na África Subsariana (ASS), onde não há uma cobertura adequada de rastreio pré-natal para a sífilis. Testes de sífilis em postos de atendimento local têm uma precisão elevada e o potencial para aumentar a cobertura de rastreio pré-natal. Contudo, há poucos dados de custo-efectividade a nível nacional para estes testes. O objectivo desta análise foi a avaliação da custo-efectividade e impacto orçamental do rastreio pré-natal da sífilis em 43 países na ASS e estimar o impacto do rastreio universal em nados-mortos, mortes neo-natais, sífilis congenital e DALY´s evitados.

**Metodos e Resultados:**

O modelo analítico de decisão da perspectiva do sistema nacional de saúde foi baseado numa sensibilidade (86%) e especificidade (99%) para os testes de bandas imunocromatográficas (BIC). Resultados clínicos de nados-mortos, mortes neo-natais e sífilis congenital de crianças nascidas de mães infectadas por sífilis foram obtidos de dados publicados. O tratamento assumiu-se consistir de três injecções de penicilina benzatina. Dados a nível nacional incluíam a prevalência pré-natal de sífilis, número de nados-vivos anual, proporção de mulheres com o mínimo de uma visita pré-natal, rendimento bruto nacional per capita e salário horário de enfermeiros estimado. Em todos os 43 países africanos subsarianos, o rastreio de sífilis é altamente custo-efectivo, com um custo por DALY evitado médio de US$11 (interval de variação: US$2-US$48). O rastreio continua altamente custo-efectivo mesmo quando a prevalência média decresce dos 3.1% actuais (intervalo de variação: 0.6%-14.0%) para 0.038% (intervalo de variação: 0.002% - 0.113%). O rastreio pré-natal universal de mulheres grávidas em clínica pode reduzir até 64,000 nados-mortos anuais, até 25,000 mortes neo-natais anuais, a incidência de sífilis congenital até 32,000 casos anuais e evitar até 2.6 milhões de DALYs com um custo directo anual médico estimado de US$20.8 milhões.

**Conclusões:** O uso de testes BIC para rastreio pré-natal da sífilis é altamente custo-efectivo na ASS. Uma redução substancial de DALYs pode ser conseguida com um impacto orçamental relativamente baixo. Na ASS, os programas pré-natais deviam ser expandidos de modo a garantir acesso ao rastreio para a sífilis usando o teste BIC.
